# Epicatechin elicits MyoD-dependent myoblast differentiation and myogenic conversion of fibroblasts

**DOI:** 10.1371/journal.pone.0175271

**Published:** 2017-04-06

**Authors:** Sang-Jin Lee, Young-Eun Leem, Ga-Yeon Go, Younhee Choi, Yoo Jin Song, Insol Kim, Do Yoon Kim, Yong Kee Kim, Dong-Wan Seo, Jong-Sun Kang, Gyu-Un Bae

**Affiliations:** 1 Research Center for Cell Fate Control, College of Pharmacy, Sookmyung Women’s University, Seoul, Republic of Korea; 2 Department of Molecular Cell Biology, Single Cell Network Research Center, Sungkyunkwan University School of Medicine, Suwon, Republic of Korea; 3 College of Pharmacy, Dankook University, Cheonan, Republic of Korea; University of California, Davis, UNITED STATES

## Abstract

Prevention of age-associated reduction in muscle mass and function is required to manage a healthy life. Supplemental (-)-Epicatechin (EC) appears to act as a potential regulator for muscle growth and strength. However, its cellular and molecular mechanisms as a potential muscle growth agent have not been studied well. In the current study, we investigated a role of EC in differentiation of muscle progenitors to gain the molecular insight into how EC regulates muscle growth. EC enhanced myogenic differentiation in a dose-dependent manner through stimulation of promyogenic signaling pathways, p38MAPK and Akt. EC treatment elevated MyoD activity by enhancing its heterodimerization with E protein. Consistently, EC also positively regulated myogenic conversion and differentiation of fibroblasts. In conclusion, EC has a potential as a therapeutic or nutraceutical remedy to treat degenerative muscle diseases or age-related muscle weakness.

## Introduction

Sarcopenia is characterized by progressive loss of muscle mass and strength [1] and linked to a gradual reduction in the regenerative capacity of the skeletal muscle stem cells, satellite cells [2]. Effective regeneration of adult skeletal muscle is largely dependent on the population, availability and functionality of satellite cells. In response to activation signals resulting from exercises or injuries, satellite cells initiate cell cycle to expand progenitors and differentiate into mature muscle cells, while small population returns to quiescence [3]. Recent studies suggest that caloric restriction can improve muscle regenerative capacity by improving satellite cell function in aging skeletal muscle accompanied by improve preservation of muscle mass and strength with aging [4, 5]. The rational approach to prevent sarcopenia is the combination of proper nutrition, possibly associated with the use of dietary supplements, and a regular exercise program [6].

(-)-Epicatechin (EC) is the stereoisomer of catechin and belongs to the group of flavanols (flavan-3-ols). EC is found in cacao beans and has the highest content of catechins among foods [7] and is made up circa 2–5% of total dry weight of green tea [8]. Anti-oxidative nutrients including EC and catechin, suppress atrogene expression in skeletal muscle cells, possibly through the inhibition of ERK signaling, resulting in prevention of unloading-mediated muscle atrophy [9]. It has been reported that EC, the main component present in dark chocolate, reduces the risk to develop cardiovascular diseases and myocardial injury [10, 11]. EC significantly promotes osteogenic proliferation, differentiation and mineralization [12]. Furthermore, the treatment of EC enhances the level of myogenic genes, such as MEF2, Myf5 and MyoD in skeletal muscles of old mice, and one week treatment with EC in humans increased muscle strength in hands [13]. However, the detailed mechanism of the positive effect of EC on muscle growth has not been examined.

In this study, we investigated the effect of EC on myoblast differentiation. EC enhances MyoD activation and myoblast differentiation through activation of key promyogenic signaling pathways, p38MAPK and Akt. In addition, EC treatment promotes the myogenic conversion of mouse embryonic fibroblasts, induced by MyoD and differentiation capacities of human rhabdomyosarcoma RD cells. Collectively, our findings suggest that EC treatment promotes myogenic differentiation via activation of key promyogenic signalings and MyoD-mediated gene expression. Thus, EC has a potential as therapeutic or nutraceutical remedy to intervene muscle weakness and muscle atrophy.

## Material and methods

### Reagents

(-)-Epicatechin (PubChem CID: 72276) was purchased from Sigma-Aldrich (St. Louis, MO). Fetal bovine serum (FBS) and Dulbecco modified Eagle’s medium (DMEM) were purchased from Thermo Scientific (Waltham, MA). Horse serum (HS) was obtained from WelGene (Daegu, Korea). For cell transfection, Lipofectamin 2000 was used (Invitrogen, Carlsbad, CA). The siRNAs for MyoD were purchased from Origene Technology (Rockville, MD). Antibodies used in this study were as following: phospho-p38MAPK (recognizing phospho-T180/-Y182 residues), phospho-Akt (recognizing the phospho-S413 residue), Akt (Cell Signaling Technology, Beverly, MA), p38MAPK, MyoD, Myogenin, E2A (Santa Cruz Biotechnology, Santa Cruz, CA), Myosin heavy chain (MHC, MF-20; the Developmental Studies Hybridoma Bank, Iowa, IA), and pan-Cadherin (Sigma-Aldrich). All other chemicals were obtained from Sigma-Aldrich.

### Cell cultures

Myoblast C2C12 cells, embryonic fibroblast 10T1/2 cells and embryonic kidney 293T cells were cultured as described previously [14, 15]. To induce differentiation of C2C12 myoblasts, cells at near confluence were switched from DMEM containing 15% FBS (fetal bovine serum; growth medium, GM) to DMEM containing 2% HS (horse serum; differentiation medium, DM) and myotube formation was observed normally at approximately 2–3 days of differentiation. The efficiency of the myotube formation was quantified by a transient differentiation assay as previously described [15]. For our experiments that involved p38MAPK inhibitors and Akt inhibitors, C2C12 cells were treated with 20 μM EC after pre-incubation with 2.5 μM SB203580 (CalBiochem, La Jolla, CA) or 1 μM LY294002 in fresh culture medium for 30 min, respectively.

Mouse embryonic fibroblasts (MEFs) isolated from C57BL/6 mice were cultured as described previously [16]. Cells were grown in F10 medium containing 20% FBS and basic fibroblast growth factor (bFGF; 100 ng/mL). For another myogenic differentiation study, 10T1/2 cells and primary MEFs were cultured in DMEM supplemented with 10% FBS in 100 mm plates, and transfected with 5 μg of MyoD or control vector (pBp). After 1–2 day incubation, 10T1/2 cells and primary MEFs were treated with 20 μM EC in 2% HS for 2 days.

### Western blot and immunoprecipitation analysis

Western blot analysis was performed as previously described [15]. Briefly, cells were lysed in cell lysis buffer (10 mM Tris-HCl, pH 7.2, 150 mM NaCl, 1 mM EDTA, 1% Triton X-100) containing complete proteinase inhibitor cocktail (Roche). Cell lysates were boiled in Laemmli sample buffer for 3 min, and then 30 μg proteins were subjected to sodium dodecyl sulfate-polyacrylamide gel electrophoresis (SDS-PAGE).

The primary antibodies used were anti-pan-Cadherin, anti-p38, anti-p-p38 (the phosphorylated, active form of p38MAPK), anti-Akt, anti-p-Akt (the phosphorylated, active form of Akt), anti-MHC, anti-Myogenin, and anti-MyoD.

For immunoprecipitation assay, 293T cells were transfected with MyoD expression vectors for 36 h. After the treatment with EC for 24 h, C2C12 or MyoD-transfected 293T cells were lysed and subjected to immunoprecipitation with anti-E2A antibodies and protein G agarose beads (Roche Diagnostics) overnight at 4°C. Beads were washed three times and resuspended in extraction buffer, and samples were analyzed by western blotting.

### Immunocytochemistry and microscopy

Immunostaining analysis for MHC expression was performed as described previously [14]. Briefly, C2C12, 10T1/2 or RD cells were fixed with 4% paraformaldehyde, permeabilized with 0.2% Triton X-100 in phosphate buffered saline, blocked, and stained with anti-MHC, followed by an Alexa Fluor 568-conjugatd secondary antibody (Molecular Probes). Images were captured and processed with a Nikon ECLIPSE TE-2000U microscope and NIS-Elements F software (Nikon, Tokyo, Japan).

### Luciferase assay

C2C12 cells were seeded in 12-well plates at a density of 4 × 10^4^ cells per well. Twenty-four hours after seeding, cells were transfected with 100 ng of 4RTK-luciferase reporter by using Lipofectamine 2000. Twenty-four hours later, transfected cells were treated with the indicated concentration of EC and differentiated in DM for 36 hours followed by luciferase activity measurement with Luciferase Reporter Assay System (Promega, Sunnyvale, CA). Experiments were performed in triplicates and repeated at least three times independently.

### Bromodeoxyuridine (BrdU) incorporation assay

C2C12 cells were cultured in 6-well plates and treated with control DMSO or 20 μM EC in GM for 24 h at 37°C. After 24 h of the treatment, 10 μM BrdU (cat. No. 550891; 10 μg/mL; BD Biosciences, San Jose, CA, USA) was added to the control DMSO- or EC-treated cells and was incubated for 30 min at 37°C. The cells were fixed and immunostained with anti-BrdU (santa Cruz Biotechnology) overnight at 4°C, followed by incubation for 2 h at room temperature with a fluorescein isothiocyanate-conjugated secondary antibody (Invitrogen). Images were captured and processed using a Nikon ECLIPSE TE-2000U microscope and NIS-Elements F software.

### Annexin V/PI staining assay

Annexin V/PI double staining was carried out with the Annexin V-FITC Apoptosis Detection kit (BD Biosciences, San Jose, CA) according to the manufacturer’s protocol, and samples were analyzed using a FACSCalibur flow cytometer (BD Biosciences) and Cell Quest 2.0 analysis software.

### Statistics

The experiments were done independently three times. The participants’ *T*-test was used to access the significance of the difference between two mean values. *P < 0.01 and **P < 0.05 were considered to be statistically significant.

## Results

### Epicatechin promotes myogenic differentiation

**Fig 1:**
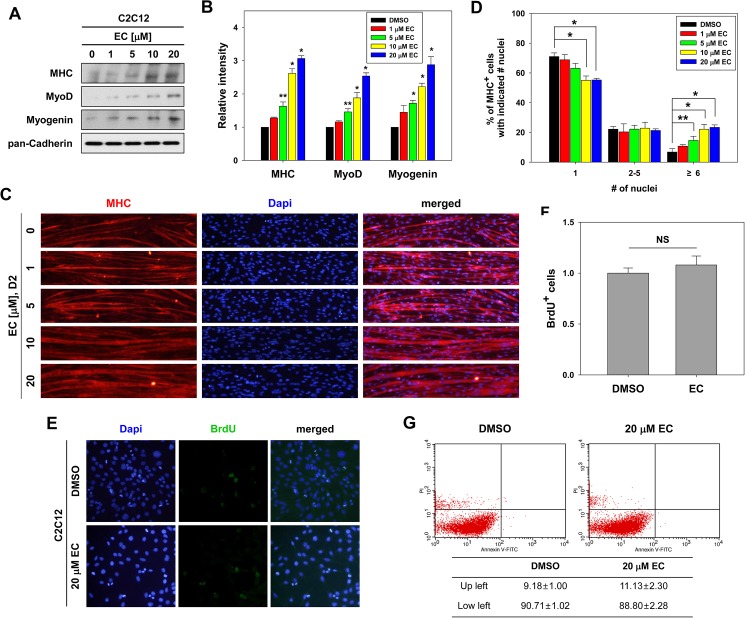
EC enhances myogenic differentiation of C2C12 cells. (A) C2C12 myoblasts were treated with indicated concentration of EC and differentiated in differentiation medium (DM) for 2 days (D2). Cell lysates were subjected to immunoblotting with antibodies to MHC, MyoD, Myogenin and pan-Cadherin as a loading control. The experiment was repeated three times with similar results. (B) Quantification of three blots from similar experiments shown in panel A. The signal intensity of MHC, MyoD and Myogenin was quantified, and the relative values were normalized to pan-Cadherin. The values of control sample were set to 1.0. Values represent the means of triplicate determinations ± 1 standard deviation (SD). *p < 0.01, **p <0.05. (C) Cells from similar experiments shown in panel A were immunostained for MHC expression (red) and DAPI to visualize nuclei (blue) to reveal myotube formation. (D) Quantification of myotube formation from data shown in panel C. Data from three independent experiments were presented as the means ± 1 SD. Asterisks indicate significant difference from the control. *P < 0.01, **P < 0.05. (E) C2C12 cells were treated with DMSO or EC for 1 day and were labeled with bromodeoxyuridine (BrdU) for 30 min followed by immunostaining with anti-BrdU antibody and DAPI staining to visualize nuclei. (F) Quantification of BrdU-positive cells presented in panel E. Data from three independent experiments were presented as the means ± 1 SD. NS, not significant. (G) Flow Cytometry analysis of Annexin V/PI. C2C12 cells were treated with DMSO or EC for 24 h and cultured in GM.

To examine the effect of EC on myoblast differentiation, C2C12 myoblasts were induced to differentiate for 2 days (D2) in the presence of vehicle DMSO or the indicated concentration of EC and assessed for its effect on myoblast differentiation by immunoblotting with muscle-specific proteins. EC-treated C2C12 myoblasts exhibited the enhanced expression of MHC, MyoD and Myogenin in a dose-dependent manner (Fig 1A and 1B). Furthermore, C2C12 myoblasts treated with EC for 2 days formed bigger MHC-positive myotubes with more nuclei, as assessed by MHC immunostaining (Fig 1C). To quantify the myotube formation, MHC-positive cells were counted as mononucleate, containing two to five nuclei, or containing six or more nuclei per myotube and plotted as percentile (Fig 1D). EC treatment reduced the proportion of mononucleate myocytes, while it substantially elevated the proportion of myotubes containing six or more nuclei in a dose-dependent manner (Fig 1D). Furthermore, C2C12 myoblasts were induced to differentiate for 2 days and then treated with 20 μM EC for additional 2 days. As results, EC treatment induced hypertrophic myotubes, resulting from Akt activation (S1 Fig). Unlike the early differentiation time point, both C2C12 cultures treated with DMSO alone or EC in differentiation medium for 6 days formed big multinucleated myotubes however EC-treated cultures formed generally larger myotubes, relative to the control cultures (S2A and S2B Fig).

Next we have assessed whether EC treatment promotes myoblast differentiation through regulation of cell proliferation or survival. To determine the effect of EC on myoblast proliferation, cells were subjected to a BrdU incorporation assay. As shown in Fig 1E and 1F, EC treatment did not overtly alter the proliferative capacity of C2C12 myoblasts, compared to DMSO-treated cells. Furthermore, EC treatment did not influence cell survival as assessed by FACS analysis (Fig 1G). These results suggest that EC enhanced differentiation of C2C12 myoblasts at a biochemical as well as a morphological level.

### Epicatechin induced activation of promyogenic kinases, p38MAPK and Akt

**Fig 2:**
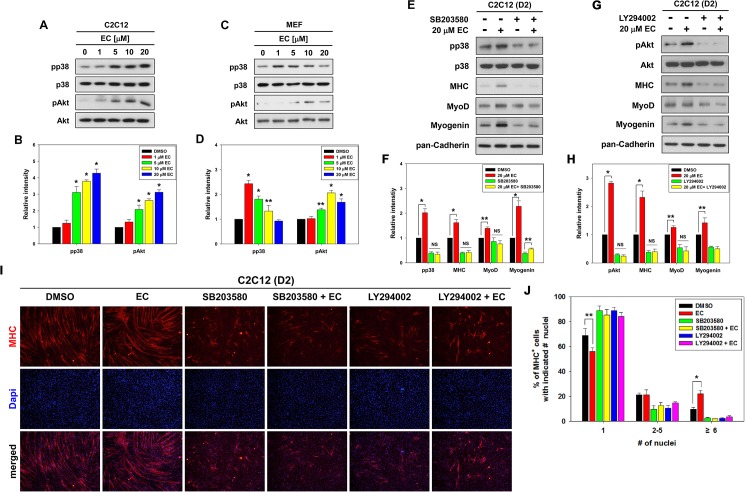
EC induces activation of p38MAPK and Akt in a dose-dependent manner. (A) C2C12 myoblasts were treated with EC and differentiated in DM for 2 days. Cell lysates were subjected to immunoblotting with antibodies to p-p38MAPK (pp38), p38MAPK (p38), p-Akt (pAkt) and Akt. The experiment was repeated three times with similar results. (B) Quantification of blots from three experiments similarly performed as shown in panel A. The relative signal intensity of pp38 and pAkt proteins to total p38 and Akt proteins, respectively was determined. The values from DMSO-treated control cells were set to 1.0. Values represent the means of triplicate determinations ± 1 SD. Significant difference from control, *P < 0.01. (C) Primary mouse MEFs were treated with EC and differentiated in DM for 2 days. Cell lysates were subjected to immunoblotting with antibodies to pp38, p38, pAkt and Akt. (D) Quantification of blots from three experiments similarly performed as shown in panel C. The relative signal intensity for pp38 and pAkt proteins to total p38 and Akt proteins, respectively was determined. The values from DMSO-treated control cells were set to 1.0. Values represent the means of triplicate determinations ± 1 SD. Significant difference from control, *P < 0.01 and **P < 0.05. (E) C2C12 myoblasts were treated with 2.5 μM SB203580 for 30 min prior to the treatment with EC, and then differentiated in DM for 2 days. Cell lysates were subjected to immunoblotting with antibodies against pp38, p38, MHC, MyoD, Myogenin and pan-Cadherin as a loading control. (F) Quantification of three blots, similarly performed as shown in panel E. The signal intensity of pp38, myogenic proteins such as MHC, MyoD and Myogenin was quantified, and the relative values were normalized to p38 and pan-Cadherin, respectively. The values of control sample were set to 1.0. Values represent the means of triplicate determinations ± 1 SD. *p < 0.01, **p <0.05. NS, not significant. (G) C2C12 myoblasts were treated with 1 μM LY294002 for 30 min prior to the treatment with EC, and then differentiated in DM for 2 days. Cell lysates were subjected to immunoblotting with antibodies against pAkt, Akt, MHC, MyoD, Myogenin and pan-Cadherin as a loading control. (H) Quantification of three blots from experiments similarly performed as shown in panel G. The signal intensity of pp38, MHC, MyoD and Myogenin was quantified, and the relative values were normalized to p38 and pan-Cadherin, respectively. The values of control sample were set to 1.0. Values represent the means of triplicate determinations ± 1 SD. *p < 0.01, **p <0.05. NS, not significant. (I) C2C12 cells from replica experiments as shown in panel E and G were immunostained for MHC expression (red) and DAPI to visualize nuclei (blue) to reveal myotube formation. (J) Quantification of myotube formation from data shown in panel I. Data from three independent experiments were presented as the means ± 1 SD. Asterisks indicate significant difference from the control. *P < 0.01, **P < 0.05.

It has been well documented that two promyogenic kinases, p38MAPK and Akt play essential roles in myoblast differentiation [17, 18]. To assess the molecular regulatory pathways of EC-mediated myoblast differentiation, C2C12 myoblasts were treated with the indicated concentration of EC and assessed for the activation status of p38MAPK and Akt by using antibodies recognizing the active phosphorylated form of p38MAPK (pp38) or Akt (pAkt) by immunoblotting. The treatment of EC increased the levels of pp38 and pAkt in a dose-dependent manner, whereas the level of total proteins stayed relatively constant (Fig 2A and 2B). In agreement with EC-treated myoblasts, primary MEFs treated with EC displayed enhanced levels of pp38 and pAkt (Fig 2C and 2D). Especially, the effective concentration of EC to activate p38MAPK in primary MEFs appears to range from 1 to 5 μM.

Next we examined whether the activities of p38MAPK and Akt are required for the EC-mediated myogenic differentiation. To do this, C2C12 myoblasts were pretreated with pharmacological inhibitors for p38MAPK (SB203580) or Akt (LY294002) for 30 minutes, respectively, prior to the treatment with EC for 48 hours in differentiation medium. Then the expression of pp38, pAkt, MHC, MyoD and Myogenin was assessed. Inhibition of p38MAPK by SB203580 resulted in the starkly reduced MHC and Myogenin levels and a mild reduction in MyoD levels, compared to the vehicle-treated cells (Fig 2E and 2F). EC treatment partially restored the expression of Myogenin in SB203580-pretreated C2C12 myoblasts, whereas EC treatment failed to restore the expression of MHC and MyoD in these cells. Similarly, Akt inhibition by LY294002 pretreatment also decreased the expression of myogenic proteins such as MHC, MyoD and Myogenin, compared to the vehicle-treated cells (Fig 2G and 2H). In addition, EC treatment failed to restore the expression of MHC, MyoD and Myogenin in Akt-inhibited cells. To further confirm, C2C12 myoblasts were induced to differentiate for 2 days in the presence of the pharmacological inhibitors and assessed by MHC immunostaining. In agreement with the previous results, inhibition of p38MAPK and Akt by SB203580 and LY294002, respectively, impaired myoblast differentiation with smaller myotube formation (Fig 2I and 2J). These results suggest that the promyogenic activity of EC is dependent on the activation of p38MAPK and Akt pathways to enhance MyoD activities and myoblast differentiation.

### Epicatechin increases MyoD activity and heterodimerization of MyoD and E proteins

**Fig 3:**
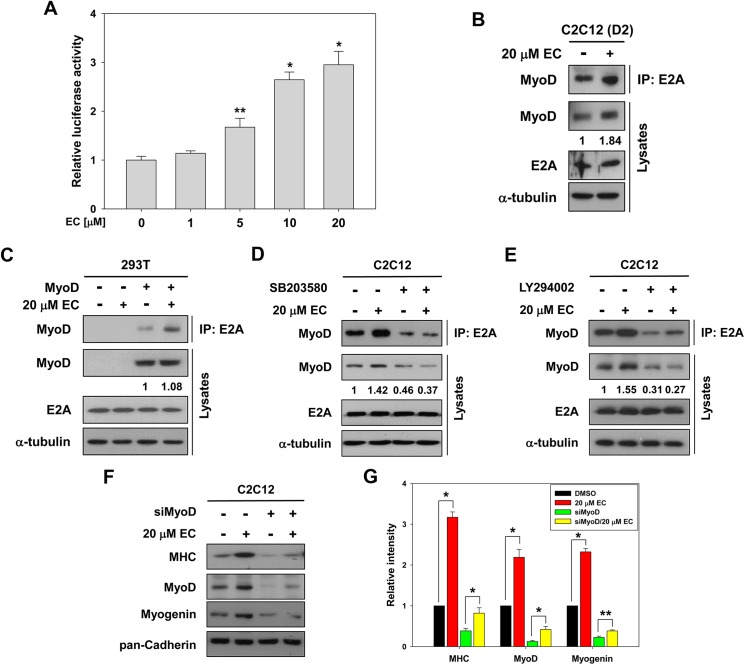
EC treatment enhances the MyoD activity and the heterodimerization of MyoD with E protein. (A) C2C12 myoblasts were transiently transfected with 4RTK-luciferases. After 24 hours, C2C12 myoblasts were treated with indicated concentration of EC and differentiated in DM for 36 hours, followed by luciferase assay. Values are means of triplicate determinants. Asterisks indicate significant difference from the control at *P < 0.01, **P < 0.05. (B) C2C12 myoblasts and (C) MyoD-transfected 293T cells were treated with DMSO or EC and subjected to immunoprecipitation with anti-E2A antibodies followed by immunoblotting analysis with anti-MyoD antibodies. Total lysates are shown as an input control for each protein. The experiment was repeated three times with similar results. (D) and (E) C2C12 myoblasts were treated with 2.5 μM SB203580 or 1 μM LY294002 for 30 min, respectively, prior to the treatment with EC, and then differentiated in DM for 2 days. Cell lysates were subjected to immunoprecipitation with anti-E2A antibodies followed by immunoblotting analysis with anti-MyoD antibodies. Total lysates are shown as input control. The experiment was repeated three times with similar results. (F) C2C12 myoblasts were transfected with MyoD siRNA or universal scrambled control siRNA, cultured to confluency and induced to differentiate for 2 days. Cell lysates were immunoblotted using antibodies to MHC, MyoD and Myogenin and to pan-Cadherin as a loading control. (G) Quantification of three blots from experiments similarly performed as shown in panel F. The signal intensity of MHC, MyoD and Myogenin was quantified, and the relative values were normalized to pan-Cadherin. The values of control sample were set to 1.0. Values represent the means of triplicate determinations ± 1 SD. *p < 0.01, **p <0.05.

To further examine the effect of EC on MyoD activation, C2C12 myoblasts were transfected with a MyoD-responsive luciferase reporter (4RTK-luc) and 24 hours later, myoblasts were transferred to the differentiation medium with DMSO alone or the indicated concentration of EC and differentiated for additional 36 hours, followed by luciferase assay. As shown in Fig 3A, EC increased the MyoD-dependent luciferase activities in a dose-dependent manner, suggesting that EC enhances the transactivation of MyoD.

Previous studies have shown that p38MAPK and Akt enhance the heterodimerization of MyoD with its partner E proteins leading to the activation of MyoD [14, 19]. Therefore we examined whether the heterodimerization of MyoD with E proteins is enhanced by EC treatment. To do this, C2C12 myoblasts were treated with vehicle or EC for 2 days in differentiation medium, followed by immunoprecipitation with antibodies to E2A and immunoblotting with MyoD antibodies. The level of E2A protein stayed constant in lysates while the level of MyoD mildly increased in EC-treated C2C12 myoblasts, relative to the vehicle-treated cells (Fig 3B). Consistently, more MyoD proteins were found in the precipitates with E2A antibodies in EC-treated C2C12 myoblasts, compared to the control cells. To further confirm EC effects on the complex formation of MyoD with E2A, 293T cells were transfected with MyoD expression vectors and treated with the vehicle or EC. Consistent with the endogenous interaction, EC treatment increased the amount of MyoD in the E2A precipitates in MyoD-transfected C2C12 myoblasts, compared to the vehicle-treated cells (Fig 3C).

To determine the role of p38MAPK and Akt in EC effects on the interaction between MyoD and E proteins, C2C12 myoblasts were pretreated with SB203580 or LY294002 for 30 minutes, respectively, prior to the treatment with EC for 48 hours in differentiation medium, followed by immunoprecipitation with antibodies to E2A and immunoblotting with MyoD antibodies. In agreement with Fig 2E and 2G, both pharmacological inhibitors decreased the heterodimerization between MyoD and E2A (Fig 3D and 3E). EC treatment failed to rescue the heterodimerization in SB203580-pretreated C2C12 myoblasts, whereas slightly restored the interaction in LY294002-pretreated C2C12 myoblasts. In Fig 3D and 3E, the total MyoD level was decreased in C2C12 cells treated with Akt or p38 inhibitors. This is likely due to the interference of auto- and cross-regulatory feedback network that functions to amplify signals for efficient myogenic differentiation [15]. In addition, we examined whether EC-induced myoblast differentiation is dependent on MyoD. To do this, C2C12 myoblasts were transfected with MyoD siRNA (siMyoD) or a universal scrambled negative control siRNA, and cultured to confluency and induced to differentiate for 2 days. As shown in Fig 3F and 3G, C2C12 myoblasts transfected with siMyoD had greatly decreased the expression levels of MyoD, MHC and Myogenin, compared to control siRNA-transfected cells. Interestingly, EC treatment partially restored the levels of MyoD, MHC and Myogenin in siMyoD-treated cells. This rescue effect is likely to be mediated by the residual amount of MyoD in C2C12/siMyoD cells. These data suggest that EC enhances MyoD activity by its heterodimerization with E proteins, likely through p38MAPK and Akt.

### Epicatechin stimulates myogenic differentiation of fibroblasts mediated by MyoD

**Fig 4:**
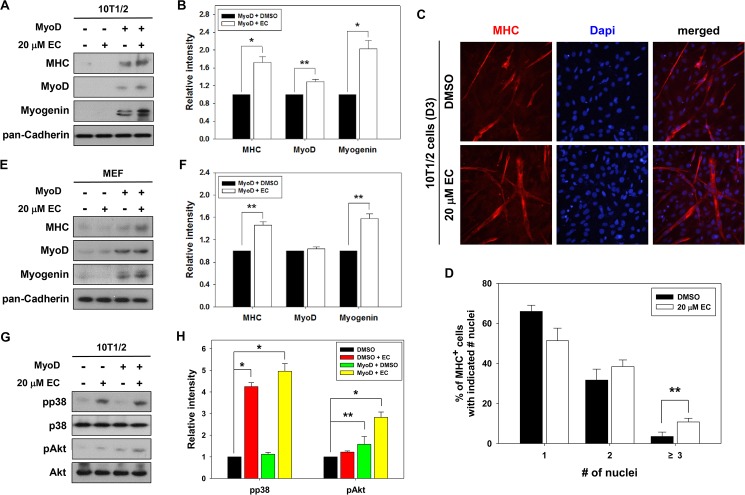
EC augments myogenic differentiation of MyoD-transfected 10T1/2 embryonic fibroblasts. (A) 10T1/2 cells were transiently transfected with control (pBp) or MyoD expression vector (pBp-MyoD) and induced to differentiate with the treatment of either 20 μM EC or DMSO for 2 days, respectively. Cell lysates were immunoblotted with antibodies to MHC, MyoD, Myogenin and pan-Cadherin as a loading control. (B) Quantification of three independent experiments, similar to data shown in panel A. The signal intensity of myogenic proteins was quantified, and normalized to the loading control pan-Cadherin. The values from control-treated MyoD-transfected cells were set to 1.0. Values represent the means of triplicate determinations ± 1 SD. Significant difference from control, *P < 0.01 and **P < 0.05. (C) 10T1/2 cells transfected with control or MyoD expression vectors were induced to differentiate with treatment of either 20 μM EC or DMSO for 3 days, followed by immunostaining for MHC expression (red) and DAPI staining (blue) to visualize the nuclei. (D) Quantification of myotube formation shown in panel C. Data from three independent experiments were presented as the means ± 1 SD. Asterisks indicate significant difference from the control at **P < 0.05. (E) Primary MEFs were transfected with control (pBp) or MyoD expression vector (pBp-MyoD) and induced to differentiate with treatment of either 20 μM EC or DMSO for 2 days, respectively. Cell lysates were immunoblotted with antibodies to MHC, MyoD, Myogenin and pan-Cadherin as a loading control. (F) Quantification of three independent experiments, similar to data shown in panel E. The signal intensity of myogenic proteins was quantified and normalized to the loading control. The values from control-treated MyoD–transfected cells were set to 1.0. Values represent the means of triplicate determinations ± 1 SD. Significant difference from control, *P < 0.01 and **P < 0.05. (G) Cell lysates shown in panel A were subjected to immunoblotting with antibodies to pp38, p38, pAkt, and Akt. (H) Quantification of three experiments performed as shown in panel G. The signal intensity of pp38, p38, pAkt, and Akt proteins were quantified, and the relative values for the phosphorylated forms to total p38 and Akt proteins were determined, respectively. The values from DMSO-treated control cells were set to 1.0. Values represent the means of triplicate determinations ± 1 SD. Significant difference from control, *P < 0.01 and **P < 0.05.

MyoD has been shown to possess the ability to convert fibroblasts into myoblasts [20]. Thus we examined whether EC enhances myogenic conversion of fibroblasts mediated by MyoD. 10T1/2 mouse embryonic fibroblast cells expressing control pBp (pBabe-puro) or pBp/MyoD were treated with DMSO or EC and induced to differentiate for 2 days, followed by immunoblot analysis and MHC immunostaining. The ectopic expression of MyoD in the vehicle-treated 10T1/2 cells induced the expression of MyoD, Myogenin and MHC, which was further enhanced by the EC treatment (Fig 4A and 4B).

MyoD-transfected 10T1/2 cells treated with EC for 3 days formed bigger and longer MHC-positive myotubes with more nuclei, compared to control-treated MyoD-transfected cells (Fig 4C). EC treatment reduced the proportion of mononucleate myocytes, while it substantially elevated the proportion of multinucleated myotubes containing three or more nuclei (Fig 4D). In agreement, EC treatment also increased the expression of MHC and Myogenin in MyoD-transfected primary MEFs, compared to the vehicle-treated cells (Fig 4E and 4F). We further investigated the activation status of p38MAPK and Akt in 10T1/2 cells. The levels of pAkt and pp38 were slightly increased in DMSO-treated 10T1/2/MyoD cells, relative to the DMSO-treated control cells (~1.2-fold and ~1.6-fold, respectively). In response to EC treatment, both control- and MyoD-expressing 10T1/2 cells exhibited a great elevation in pp38 levels, while only 10T1/2/MyoD cells showed a robust Akt activation when treated with EC, compared to the control-expressing cells. These results suggest that EC treatment augments MyoD-mediated myogenic differentiation in fibroblasts through p38MAPK and Akt activation.

### Epicatechin enhances myogenic differentiation of human rhabdomyosarcoma cells

**Fig 5:**
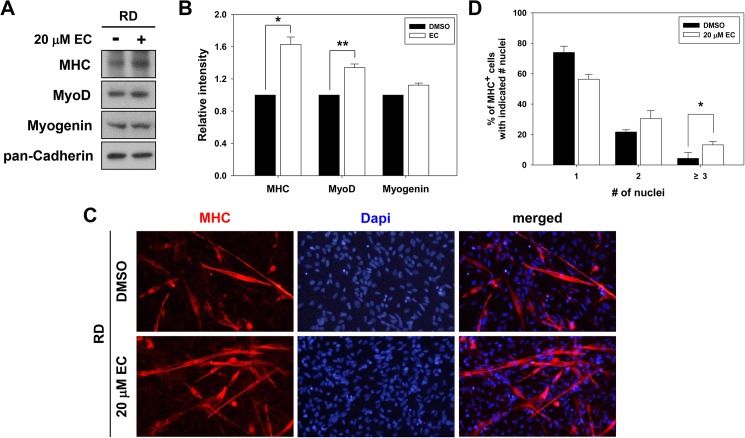
EC enhances myogenic differentiation of human RD cells. (A) RD cells were induced to differentiate with treatment of either EC or DMSO for 72 hours. Cell lysates were subjected to immunoblotting with antibodies to MHC, MyoD, Myogenin and pan-Cadherin as a loading control. The experiment was repeated three times with similar results. (B) Quantification of three independent experiments performed as shown in panel A. The intensity of myogenic-specific proteins was quantified, and the values from DMSO were set to 1.0. Data from three independent experiments were presented as the means ± 1 SD. Significant difference from control, *P < 0.01 and **P < 0.05. (C) Cells from panel A were stained for MHC expression (red) and with DAPI to stain nuclei (blue) to reveal myotube formation. (D) Quantification of myotube formation in experiments shown in panel (C). Data from three independent experiments were presented as the means ± 1 SD. Asterisks indicate significant difference from the control at *P < 0.01, **P < 0.05.

Rhabdomyosarcoma (RD) is a malignant form of cancer that arises from skeletal muscle cells with impaired differentiation characteristics [21]. We have tested whether EC can improve the differentiation capacity of human rhabdomyosarcoma cells. Human RD cells were treated with EC for 72 hours and subjected to immunoblotting for the expression of MHC, MyoD and Myogenin. As shown in Fig 5A and 5B, EC treatment enhanced the expression of MHC and MyoD, compared to the vehicle-treated RD cells, while the expression of Myogenin was not altered by EC treatment. Furthermore, EC treatment also improved myotube formation and elevated the proportion of larger myotubes with more nuclei per myotube, compared to vehicle-treated RD cells (Fig 5C and 5D). Furthermore, control- or EC-treated RD cells induced to differentiate for 6 days were all positive for MHC, and EC-treated RD cells formed more multinucleated myotubes, relative to DMSO-treated cells (S2C and S2D Fig). These results indicate that EC can ameliorate the differentiation capacity of human RD cells.

## Discussion

There have been many efforts to identify effective pharmacological or nutritional supplements to counteract the progressive loss of muscle mass and to enhance the muscle strength in pathological conditions or muscle aging. In this study, we have tried to investigate the effect and molecular mechanism of EC as a compound for improving muscle differentiation. The positive role of EC in the regulation of muscle growth and differentiation has been previously referred by Ramirez-Sanchez group [13]. However, the molecular mechanism by which EC modulates myogenic differentiation was unclear. In the current study, our results demonstrate that EC promotes myoblast differentiation through activation of p38MAPK and Akt which in turn activates MyoD and myogenic differentiation. p38MAPK and Akt play key roles in myogenic differentiation through augmenting MyoD-mediated muscle-specific gene expression, such as MHC and Myogenin [15, 22–24] and these myogenic signaling pathways are involved in a positive feedback network that amplifies and maintains the myogenic phenotype [25].

The involvement of p38MAPK and AKT pathways in EC-mediated myoblast differentiation are multi-fold. EC treatment enhances activation of p38MAPK and Akt in a dose-dependent manner and inhibition of p38MAPK and Akt abrogates the promyogenic effects of EC treatment in C2C12 cells. Previous studies have shown that p38MAPK enhances the functional heterodimer formation of MyoD with E protein partner which can be directly phosphorylated by p38MAPK [26]. In line with this notion, EC treatment enhanced p38MAPK activation and the heterodimerization of MyoD with E proteins in C2C12 myoblasts as well as in MyoD-expressing 293T cells. Furthermore, inhibition of p38MAPK by SB203580 treatment abrogated the enhancing effect of EC treatment on MyoD-E protein heterodimerization. The promyogenic effect of EC appears to be mediated by MyoD, since MyoD depletion revoked the enhanced expression of MHC and Myogenin. Similar effects were further observed in 10T1/2 and primary MEF cells expressing MyoD. The stimulatory effect of EC on myogenic conversion of non-myogenic cells might be beneficial to improve the muscle regeneration in aging or dystrophic environment.

The effect of the long-term EC treatment on the hypertrophic myotube formation could be mediated by Akt signaling pathway. The Akt pathway plays a major role in regulation of muscle hypertrophy [27], and overexpression of a constitutively active form of serine/threonine kinase Akt leads to a rapid increase in protein synthesis and significant muscle growth [28]. Interestingly, the treatment of EC at the early differentiation stage elicits activation of both p38MAPK and Akt, while the treatment of EC at the late differentiation stage mainly results in Akt activation, which might be contributing to myotube growth. Considering that the progressive loss of muscle and elevated muscle fibrosis correlates with aberrant muscle repair linked with age-related muscle atrophy [29, 30], the enhancing myogenic capacity of muscle precursor cells by EC might be an effective prevention to improve muscle regeneration. Thus EC might be a novel effective nutritional supplement for facilitating muscle hypertrophy, and has a potential use in the treatment of muscle atrophy related to normal aging or pathological conditions.

## Conclusions

Our study provides a mechanistic framework for understanding how EC promotes muscle growth by enhancing MyoD activities and myoblast differentiation. Furthermore EC also augments myogenic conversion of fibroblasts, suggesting that EC has a potential as a nutraceutical remedy to treat muscle weakness and muscle loss.

## Supporting information

S1 Fig EC treatment in later stage of myoblast differentiation enhances myotube growth. (TIF)

S2 Fig Prolonged EC treatment enhances myotube growth in C2C12 cells and augments myotube formation of RD cells. (TIF)

## References

[1] Cruz-Jentoft AJ, Baeyens JP, Bauer JM, Boirie Y, Cederholm T, Landi F, et al Sarcopenia: European consensus on definition and diagnosis: Report of the European Working Group on Sarcopenia in Older People. Age Ageing. 2010;39(4):412–23. PubMed Central PMCID: PMCPMC2886201. doi: 10.1093/ageing/afq034 20392703 10.1093/ageing/afq034 PMC2886201

[2] Sousa-Victor P, Munoz-Canoves P. Regenerative decline of stem cells in sarcopenia. Mol Aspects Med. 2016. 10.1016/j.mam.2016.02.002 26921790

[3] Charge SB, Rudnicki MA. Cellular and molecular regulation of muscle regeneration. Physiol Rev. 2004;84(1):209–38. doi: 10.1152/physrev.00019.2003 14715915 10.1152/physrev.00019.2003

[4] Cerletti M, Jang YC, Finley LW, Haigis MC, Wagers AJ. Short-term calorie restriction enhances skeletal muscle stem cell function. Cell Stem Cell. 2012;10(5):515–9. PubMed Central PMCID: PMCPMC3561899. doi: 10.1016/j.stem.2012.04.002 22560075 10.1016/j.stem.2012.04.002 PMC3561899

[5] Colman RJ, Beasley TM, Allison DB, Weindruch R. Attenuation of sarcopenia by dietary restriction in rhesus monkeys. J Gerontol A Biol Sci Med Sci. 2008;63(6):556–9. PubMed Central PMCID: PMCPMC2812805. 18559628 10.1093/gerona/63.6.556 PMC2812805

[6] Rondanelli M, Faliva M, Monteferrario F, Peroni G, Repaci E, Allieri F, et al Novel insights on nutrient management of sarcopenia in elderly. Biomed Res Int. 2015;2015:524948 PubMed Central PMCID: PMCPMC4326274. doi: 10.1155/2015/524948 25705670 10.1155/2015/524948 PMC4326274

[7] Kwik-Uribe C, Bektash RM. Cocoa flavanols—measurement, bioavailability and bioactivity. Asia Pac J Clin Nutr. 2008;17 Suppl 1:280–3. 18296356

[8] Wang S, Noh SK, Koo SI. Green tea catechins inhibit pancreatic phospholipase A(2) and intestinal absorption of lipids in ovariectomized rats. J Nutr Biochem. 2006;17(7):492–8. doi: 10.1016/j.jnutbio.2006.03.004 16713229 10.1016/j.jnutbio.2006.03.004

[9] Hemdan DI, Hirasaka K, Nakao R, Kohno S, Kagawa S, Abe T, et al Polyphenols prevent clinorotation-induced expression of atrogenes in mouse C2C12 skeletal myotubes. J Med Invest. 2009;56(1–2):26–32. 19262011 10.2152/jmi.56.26

[10] Buijsse B, Weikert C, Drogan D, Bergmann M, Boeing H. Chocolate consumption in relation to blood pressure and risk of cardiovascular disease in German adults. Eur Heart J. 2010;31(13):1616–23. doi: 10.1093/eurheartj/ehq068 20354055 10.1093/eurheartj/ehq068

[11] Ramirez-Sanchez I, Maya L, Ceballos G, Villarreal F. (-)-epicatechin activation of endothelial cell endothelial nitric oxide synthase, nitric oxide, and related signaling pathways. Hypertension. 2010;55(6):1398–405. PubMed Central PMCID: PMCPMC2874202. doi: 10.1161/HYPERTENSIONAHA.109.147892 20404222 10.1161/HYPERTENSIONAHA.109.147892 PMC2874202

[12] Zeng X, Tian J, Cai K, Wu X, Wang Y, Zheng Y, et al Promoting osteoblast differentiation by the flavanes from Huangshan Maofeng tea is linked to a reduction of oxidative stress. Phytomedicine. 2014;21(3):217–24. doi: 10.1016/j.phymed.2013.08.026 24075209 10.1016/j.phymed.2013.08.026

[13] Gutierrez-Salmean G, Ciaraldi TP, Nogueira L, Barboza J, Taub PR, Hogan MC, et al Effects of (-)-epicatechin on molecular modulators of skeletal muscle growth and differentiation. J Nutr Biochem. 2014;25(1):91–4. PubMed Central PMCID: PMCPMC3857584. doi: 10.1016/j.jnutbio.2013.09.007 24314870 10.1016/j.jnutbio.2013.09.007 PMC3857584

[14] Bae GU, Kim BG, Lee HJ, Oh JE, Lee SJ, Zhang W, et al Cdo binds Abl to promote p38alpha/beta mitogen-activated protein kinase activity and myogenic differentiation. Mol Cell Biol. 2009;29(15):4130–43. PubMed Central PMCID: PMCPMC2715815. doi: 10.1128/MCB.00199-09 19470755 10.1128/MCB.00199-09 PMC2715815

[15] Bae GU, Lee JR, Kim BG, Han JW, Leem YE, Lee HJ, et al Cdo interacts with APPL1 and activates Akt in myoblast differentiation. Mol Biol Cell. 2010;21(14):2399–411. PubMed Central PMCID: PMCPMC2903669. doi: 10.1091/mbc.E09-12-1011 20484574 10.1091/mbc.E09-12-1011 PMC2903669

[16] Cole F, Krauss RS. Microform holoprosencephaly in mice that lack the Ig superfamily member Cdon. Curr Biol. 2003;13(5):411–5. 12620190 10.1016/s0960-9822(03)00088-5

[17] Serra C, Palacios D, Mozzetta C, Forcales SV, Morantte I, Ripani M, et al Functional interdependence at the chromatin level between the MKK6/p38 and IGF1/PI3K/AKT pathways during muscle differentiation. Mol Cell. 2007;28(2):200–13. PubMed Central PMCID: PMCPMC2693200. doi: 10.1016/j.molcel.2007.08.021 17964260 10.1016/j.molcel.2007.08.021 PMC2693200

[18] Simone C, Forcales SV, Hill DA, Imbalzano AN, Latella L, Puri PL. p38 pathway targets SWI-SNF chromatin-remodeling complex to muscle-specific loci. Nat Genet. 2004;36(7):738–43. doi: 10.1038/ng1378 15208625 10.1038/ng1378

[19] Wilson EM, Rotwein P. Selective control of skeletal muscle differentiation by Akt1. J Biol Chem. 2007;282(8):5106–10. doi: 10.1074/jbc.C600315200 17218321 10.1074/jbc.C600315200

[20] Tapscott SJ, Davis RL, Thayer MJ, Cheng PF, Weintraub H, Lassar AB. MyoD1: a nuclear phosphoprotein requiring a Myc homology region to convert fibroblasts to myoblasts. Science. 1988;242(4877):405–11. 3175662 10.1126/science.3175662

[21] Tonin PN, Scrable H, Shimada H, Cavenee WK. Muscle-specific gene expression in rhabdomyosarcomas and stages of human fetal skeletal muscle development. Cancer Res. 1991;51(19):5100–6. 1717137

[22] Kaneko S, Feldman RI, Yu L, Wu Z, Gritsko T, Shelley SA, et al Positive feedback regulation between Akt2 and MyoD during muscle differentiation. Cloning of Akt2 promoter. J Biol Chem. 2002;277(26):23230–5. doi: 10.1074/jbc.M201733200 11948187 10.1074/jbc.M201733200

[23] Bergstrom DA, Penn BH, Strand A, Perry RL, Rudnicki MA, Tapscott SJ. Promoter-specific regulation of MyoD binding and signal transduction cooperate to pattern gene expression. Mol Cell. 2002;9(3):587–600. 11931766 10.1016/s1097-2765(02)00481-1

[24] Krauss RS, Cole F, Gaio U, Takaesu G, Zhang W, Kang JS. Close encounters: regulation of vertebrate skeletal myogenesis by cell-cell contact. J Cell Sci. 2005;118(Pt 11):2355–62. doi: 10.1242/jcs.02397 15923648 10.1242/jcs.02397

[25] Ludolph DC, Konieczny SF. Transcription factor families: muscling in on the myogenic program. FASEB J. 1995;9(15):1595–604. 8529839 10.1096/fasebj.9.15.8529839

[26] Neuhold LA, Wold B. HLH forced dimers: tethering MyoD to E47 generates a dominant positive myogenic factor insulated from negative regulation by Id. Cell. 1993;74(6):1033–42. 7691411 10.1016/0092-8674(93)90725-6

[27] Sandri M, Barberi L, Bijlsma AY, Blaauw B, Dyar KA, Milan G, et al Signalling pathways regulating muscle mass in ageing skeletal muscle: the role of the IGF1-Akt-mTOR-FoxO pathway. Biogerontology. 2013;14(3):303–23. doi: 10.1007/s10522-013-9432-9 23686362 10.1007/s10522-013-9432-9

[28] Stitt TN, Drujan D, Clarke BA, Panaro F, Timofeyva Y, Kline WO, et al The IGF-1/PI3K/Akt pathway prevents expression of muscle atrophy-induced ubiquitin ligases by inhibiting FOXO transcription factors. Mol Cell. 2004;14(3):395–403. 15125842 10.1016/s1097-2765(04)00211-4

[29] Jackman RW, Kandarian SC. The molecular basis of skeletal muscle atrophy. Am J Physiol Cell Physiol. 2004;287(4):C834–43. doi: 10.1152/ajpcell.00579.2003 15355854 10.1152/ajpcell.00579.2003

[30] Mann CJ, Perdiguero E, Kharraz Y, Aguilar S, Pessina P, Serrano AL, et al Aberrant repair and fibrosis development in skeletal muscle. Skelet Muscle. 2011;1(1):21 PubMed Central PMCID: PMCPMC3156644. doi: 10.1186/2044-5040-1-21 21798099 10.1186/2044-5040-1-21 PMC3156644

