# Supplementary figures and images for: Epicatechin elicits MyoD-dependent myoblast differentiation and myogenic conversion of fibroblasts
Source: PLoS One. 2017 Apr 6;12(4):e0175271. doi: 10.1371/journal.pone.0175271 (PMC5383328; doi:10.1371/journal.pone.0175271)

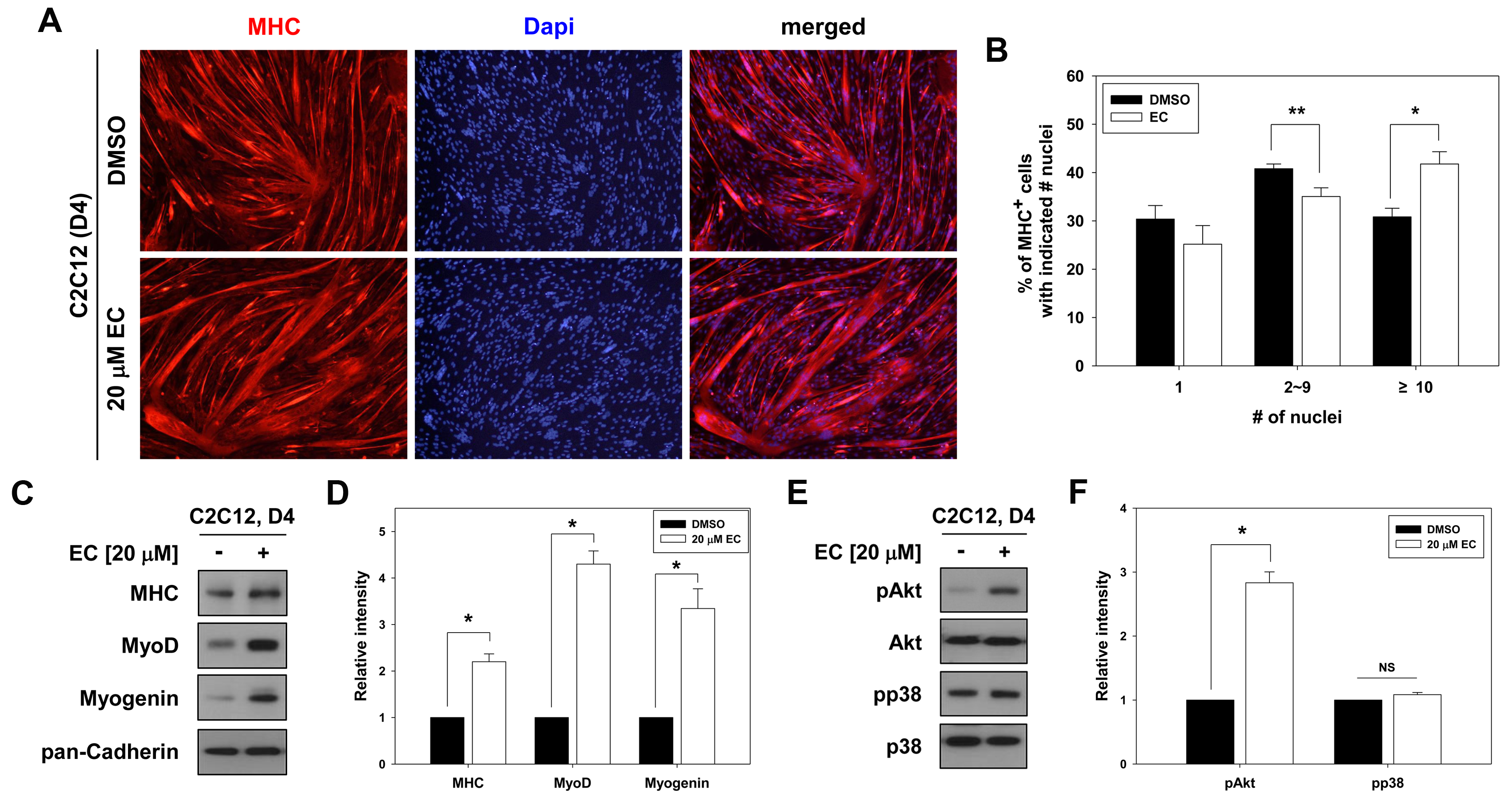

Supplement: S1 Fig — (TIF) [file pone.0175271.s001.tif]

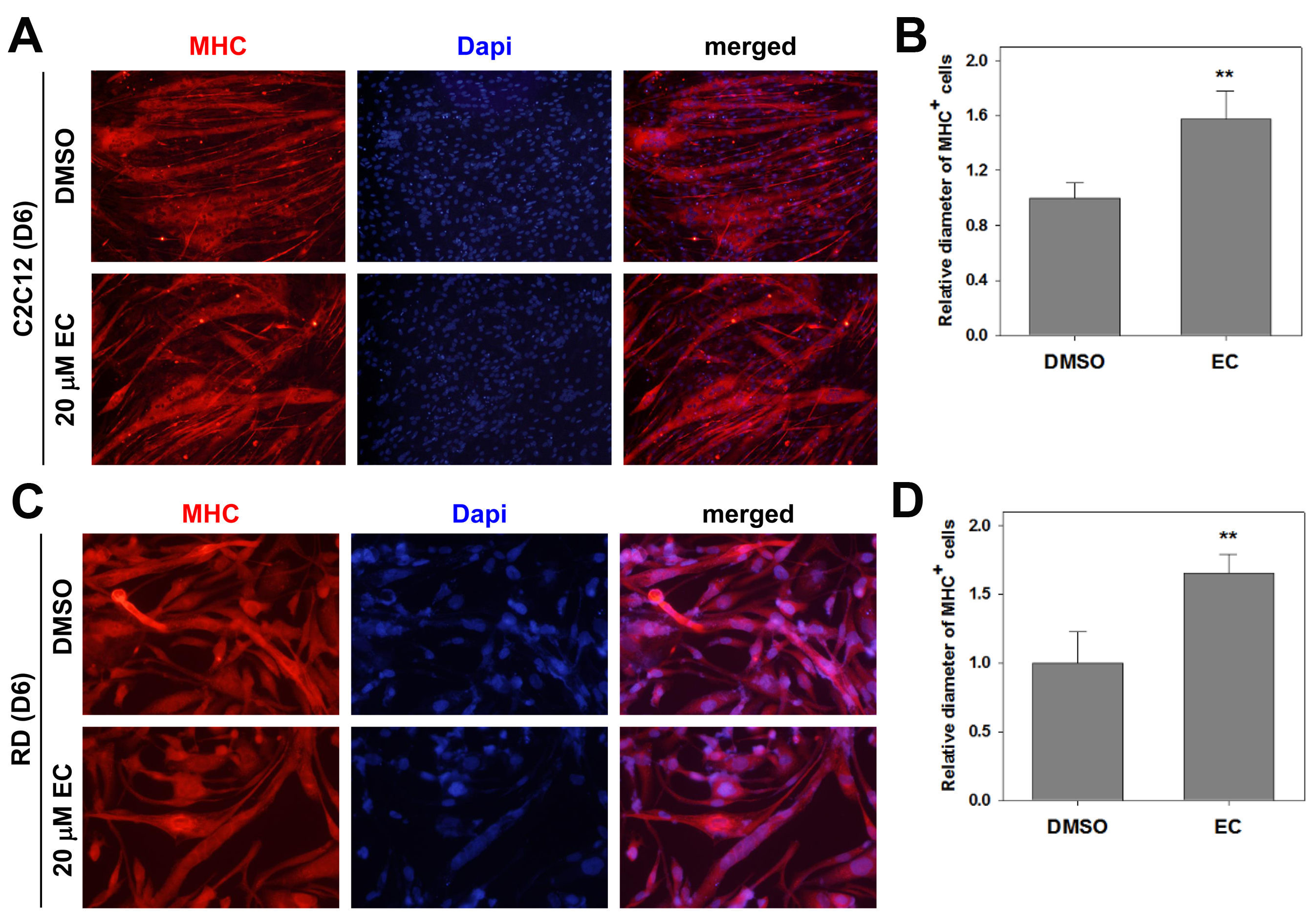

Supplement: S2 Fig — (TIF) [file pone.0175271.s002.tif]
